# A Quantitative Phase Analysis by Neutron Diffraction of Conventional and Advanced Aluminum Alloys Thermally Conditioned for Elevated-Temperature Applications

**DOI:** 10.3390/ma17174311

**Published:** 2024-08-30

**Authors:** Jordan Roger Kozakevich, Dimitry Sediako, David Weiss, Sven C. Vogel

**Affiliations:** 1 High-Performance Powertrain Materials Laboratory, University of British Columbia—Okanagan, 1137 Alumni Ave., Kelowna, BC V1V 1V7, Canada; dimitry.sediako@ubc.ca; 2 Eck Industries, 1602 N 8th St., Manitowoc, WI 54220, USA; david.weiss@eckindustries.com; 3 Loukus Technologies, 58390 Centennial Number 6 Road, Calumet, MI 49913, USA; 4 Materials Science and Technology Division, Los Alamos National Laboratory, Los Alamos, NM 87545, USA

**Keywords:** aluminum, cerium, neutron diffraction, quantitative phase analysis, high temperatures

## Abstract

As the issue of climate change becomes more prevalent, engineers have focused on developing lightweight Al alloys capable of increasing the power density of powertrains. The characterization of these alloys has been focused on mechanical properties and less on the fundamental response of microstructures to achieve these properties. Therefore, this study assesses the quality of the microstructure of two high-temperature Al alloys (A356 + 3.5RE and Al-8Ce-10Mg), comparing them to T6 A356. These alloys underwent thermal conditioning at 250 and 300 °C for 200 h. Time-of-flight neutron diffraction experiments were performed before and after conditioning. The phase evolution was quantified using Rietveld refinement. It was found that the Si phase grows significantly (13–24%) in T6 A356, A356 + 3.5RE, and T6 A356 + 3.5RE alloys, which is typically correlated with a reduction in mechanical properties. Subjecting the A356 3.5RE alloy to a T6 heat treatment stabilizes the orthorhombic Al_4_Ce_3_Si_6_ and monoclinic β-Al_5_FeSi phases, making them resistant to thermal conditioning. These two phases are known for enhancing mechanical properties. Additionally, the T6 treatment reduced the vol.% of the cubic Al_20_CeTi_2_ and hexagonal ᴨ-Al_9_FeSi_3_Mg_5_ phases by 13% and 23%, respectively. These phases have detrimental mechanical properties. The Al-8Ce-10Mg alloy cubic β-Al_3_Mg_2_ phase showed significant growth (82–101%) in response to conditioning, while the orthorhombic Al_11_Ce_3_ phase remained stable. The growth of the beta phase is known to decrease the mechanical properties of this alloy. These efforts give valuable insight into how these alloys will perform and evolve in demanding high-temperature environments.

## 1. Introduction

Advanced aluminum (Al) alloys capable of operating in elevated-temperature environments, a subject of ongoing research and development in the transportation industry, pose a significant design challenge for material engineers. Conventional Al alloys are renowned for their high strength-to-weight ratio, low density, excellent creep resistance, and good castability, making them a compelling choice for various applications [1,2,3,4]. Al-Si conventional alloys, such as A356 and B319, have emerged as key players in the transportation industry, particularly in the aerospace and automotive sectors, by providing a solution to reduce weight and combat climate change by reducing greenhouse gas emissions [3,5,6,7,8]. These clear benefits have encouraged the use of Al-Si-based alloys for geometrically complex cast automotive components, such as transmission cases, engine blocks, and turbines. However, the automotive industry’s needs are evolving. Internal combustion engines are being phased out because they emit significant amounts of greenhouse gas emissions, contributing to climate change. One of the most promising technologies to replace internal combustion engines is replacing their fuel source with hydrogen. Hydrogen-fueled internal combustion engines share similar manufacturing and infrastructure requirements but with the added benefit of producing H_2_O as a combustion byproduct rather than harmful greenhouse gases. However, the ignition point of hydrogen (587 °C) is significantly higher than gasoline (260 °C). Additionally, hydrogen internal combustion engines require a compression ratio closer to the lower end of diesel engines, much higher than conventional spark-ignition ones. This additional temperature and pressure pose a challenging material problem [9,10]. Most engine blocks today are made up of Al alloys for the benefits previously stated. Moreover, Al alloys are some of the most resistant materials to gaseous H_2_ embrittlement, which would be good for such applications. However, in the conversion to hydrogen internal combustion engines, a significant problem arises from the microstructural thermal instability of conventional Al alloys.

Fundamentally, Al-Si-based alloys rely on intermetallics such as cubic Mg_2_Si and Si phases for their strengthening benefits [3,11,12,13]. Heat treatments can manipulate the volume percentages (vol.%) and morphologies of these phases for additional strengthening benefits, one of the most common being a T6 condition (solutionizing + peak aging) [14]. However, this manipulated microstructure also limits the ability of Al-Si-based alloys to maintain their mechanical properties in elevated-temperature applications. In operating environments above 200 °C, Mg_2_Si and Si phases have limited coarsening resistance [11,15]; therefore, most internal combustion engines today have an operating temperature limit under 200 °C [16]. Once these phases begin to coarsen, the alloy loses its dispersoid and solid solution strengthening effects as Mg and Si migrate from the solid solution and coarsen the Si and Mg_2_Si phases. The coarsening is typically subtle but can be significantly detrimental to mechanical properties. For example, Stroh et al. [17] tested a T6 A356 alloy in tension at 250 °C and found that the alloy exhibited an ultimate tensile strength (UTS) and yield strength (YS) of just 66 and 63 MPa, respectively. When the testing temperature was increased to 300 °C, the UTS and YS were reduced by over 50% to 31 and 25 MPa, respectively. At ambient temperature, an A356 alloy with a T6 heat treatment typically exhibited a UTS and YS of 234 and 165 MPa, respectively [18]. This inability of conventional Al alloys to perform adequately when exposed to elevated temperatures severely limits their applicability for hydrogen internal combustion engines; the high ignition point of H_2_ will most certainly lead to a significant increase in operating temperatures above 200 °C.

Therefore, over the last decade, material scientists have found that some of the research in the 20th century on alloying Al with rare-earth (RE) elements can help with the issue of insufficient thermal stability in Al alloys. For example, in 1999, Belov et al. [19] conducted elevated-temperature tensile tests on several Al alloys with cerium (Ce, a light rare-earth metal) and nickel (Ni) additions. Targeting 350 °C, they found that an Al-12Ce-5Ni (wt. %) alloy had a 75% increase in UTS over an A339 (Al-12.0Si-2.3Cu-1.0Mg in wt. %), which was conventionally used as a piston alloy then. The enhanced thermal stability was attributed to the thermally stable binary eutectics (orthorhombic Al_4_Ce, now known as Al11Ce_3_ and Al_3_Ni). These findings have been the basis of most modern-day research focused on alloying Al with rare-earth (RE) elements [1,7,20,21,22,23,24,25,26,27,28,29,30]. For example, Aghaie et al. [31] found that adding 0.1 wt. % Ce to a B319 alloy increased the material’s UTS and YS at 250 °C by 7% and 14%, respectively. The increases in UTS and YS at 250 °C were attributed to the formation of Al_3_Ce_4_Si_6_ and AlCeSi_2_ phases. The added Ce chemical also refined the eutectic Si phase, which helped enhance the UTS and YS at 250 °C.

Such benefits are also found with much higher additions of RE elements in conventional Al alloys. For example, Stroh et al. [1,17], in a two-part study, investigated the alloy A356 with 3.5% RE mischmetal (~50% cerium, 26% lanthanum, 16 wt. % neodymium, and 6 wt. % praseodymium) additions into an A356 alloy. In the first study, the authors of [17] found that casting A356 with 3.5 wt. % additions of RE and subjecting the alloy to a T6 heat treatment resulted in a 133% increase in YS at 250 °C. Additionally, the modified alloy experienced a 158% increase in YS at 300 °C over the conventional T6 A356 alloy. The increased mechanical properties were attributed to the thermally stable orthorhombic AlSiRE and cubic Al_20_Ti_2_RE phases as well as the spheroidization of the Si phase after heat treatment. The second study [1] took the same A356 alloy with 3.5 wt. % RE mischmetal and refined the ratio of magnesium–manganese (Mg-Mn), two elements already present in A356. The Mg content decreased from 0.49 to 0.25 wt. % while the Mn content increased from 0.10 to 0.41 wt. %. This compositional modification refined the harmful iron-bearing intermetallics, resulting in increases in the UTS, YS, and modulus of elasticity of 9%, 14%, and 10%, respectively, at 250 °C compared to the unmodified A356 + 3.5% RE. One of the more profound insights of this second study is how Ce interacts in complex Al alloy systems.

Studies like these are prompting research into utilizing the Al-Ce alloying system as a base for next-generation powertrain alloys [5,7,18,23,32,33]. In the last 5 years, extensive research has gone into the fundamentals of the Al-Ce binary system to understand the platform from which a new alloying system can be structured. The solidification characteristics and phase analysis of hypoeutectic [20,25,34], eutectic [25,35,36,37], and hypereutectic [20,25,36,38,39] binary alloy compositions revealed that the orthorhombic Al_11_Ce_3_ phase has excellent castability and thermal stability up to 500 °C. In a hypereutectic composition, primary Al_11_Ce_3_ formation occurs. If the composition of Ce exceeds 16 wt. %, the primary phase begins to crack due to the large coefficient of thermal expansion discrepancy [33]. Therefore, most studies focus on the near-eutectic composition to avoid any possible decrease in mechanical properties. These eutectic alloys have a lamellae eutectic Al-Al_11_Ce_3_ phase that can retain up to 80% of its hardness when exposed to 500 °C for 168 h [24,35,37]. For comparison, the Al-Si eutectic composition only retains ~50% of its hardness when exposed to the same experiment [37]. Weiss et al. [40] took the Al-Ce binary system further and tested two ternary Al-Ce alloys with magnesium (Mg) additions, each with 8 wt. % Ce but varying Mg content (7 vs. 10 wt. %). Both alloys were tested at ambient temperature and 260 °C. The alloy containing 7 wt. % Mg exhibited a UTS and YS of 195 and 151 MPa, respectively. The alloy with 10 wt. % Mg exhibited a UTS and YS of 227 and 186 MPa, respectively. When tested at 260 °C, the 7 wt. % Mg alloy retained 69% of its UTS and 80% of its YS. The 10 wt. % Mg alloy retained 60% of its UTS and 70% of its YS. An important point to note is that these alloys were not conditioned (i.e., exposed to 260 °C for an extended period) before testing.

Thermal conditioning (sometimes attached to terms like ‘over-aging’) is crucial in the elevated-temperature testing of Al alloys. If an alloy is not thermally conditioned, the microstructure is not stabilized. Testing an instability in a microstructure leads to mechanical properties that do not accurately represent the alloy at the intended operating temperature. This effect becomes even more critical when the application requires long-term exposure to elevated temperatures. Studies have shown that thermally conditional Al-Si-based alloys between 15 min and 200 h can lead to a 20–50% decrease in mechanical properties compared to non-conditioned alloys [2,41].

The investigation into the microstructures of these alloys before and after thermal conditioning has been a sparse area in research. Even studies that do promote the concept of thermal conditioning tend to focus more on the mechanical properties and typically only assess the microstructure via surface techniques such as optical microscopy, scanning electron microscopy (SEM), X-ray diffraction (XRD), and electron backscatter diffraction (EBSD). Although these techniques are conventional in the materials engineering world, their assessment of quantitatively analyzing bulk phases of a material can be skewed by surface preparation, texture within the material, and the statistical reliability of the area chosen to present the microstructure by the researcher. These drawbacks can be significantly improved using time-of-flight neutron diffraction for bulk phase analysis. Utilizing time-of-flight neutron diffraction allows for the account of how the texture of each phase, even in small amounts, contributes to changes in intensity peaks [42]. This account of texture results in a much more accurate Rietveld refinement and a much more accurate bulk phase analysis [43].

Therefore, this study aims to take the most promising advanced Al alloys (A356 + 3.5RE and Al-8Ce-10Mg) and compare them to a conventional T6 A356 via a quantitative phase analysis (QPA) using time-of-flight neutron diffraction. This QPA study will identify the growth and dissolution effects that thermal conditioning has on stabilizing the microstructure of these alloys at 250 and 300 °C, two desirable temperatures sought after for operating temperatures of hydrogen internal combustion engines.

## 2. Materials and Methods

This section provides relevant details on the materials, sample manufacturing parameters, neutron diffraction experimentation specifics, Rietveld analysis (using MAUD, version 2.9993), and ThermoCalc^TM^ software packages (2024a, Thermo-Calc Software, Solna, Sweden) used to characterize the alloys’ microstructures. These details make this study reproducible for others interested in using it to expand the concepts of the characterization of other materials for engineering purposes.

### 2.1. Materials and Sample Preparation

This study investigated three alloys of interest: A356, A356 + 3.5RE, and Al-8Ce-10Mg. These alloys were cast at Eck Industries in Manitowoc, WI, USA. A356 ingots were used to cast the A356 samples and fabricate the A356 + 3.5% RE alloy. The 356 + 3.5% RE alloy was fabricated by introducing the RE mischmetal to an A356 melt before mixing with an impeller at 250 RPM, similar to Stroh et al. in [17]. The alloy was degassed with argon for 20 min before being poured into an ASTM permanent tensile mould preheated to 400 °C. The Al-8Ce-10Mg alloy was cast from prepared ingots that were melted, degassed, and poured, replicating the same process as the A356 and A356 + 3.5RE alloys. The composition of each alloy is shown in Table 1.

All three alloys were cast as tensile bars at Eck Industries and provided to the High-Performance Powertrain Materials (HPPM) laboratory at the University of British Columbia. At the HPPM laboratory, one A356 tensile bar and one A356 + 3.5RE tensile bar were T6 heat treated. The T6 heat treatments consisted of solution annealing at 538 °C (1000 °F) for 8 h, quenching in water heated to 78 °C (172 °F), held at ambient temperature for 12 h, and then aged at 154 °C (310 °F) for 4 h, after which the alloys were cooled naturally to room temperature.

At the HPPM laboratory, all as-cast (AC) and T6 alloys were machined into cylindrical samples 10 mm in diameter and 12 mm tall from the gauge section of the tensile bars. One sample from each alloy was thermally conditioned at 250 °C, while another was conditioned at 300 °C for 200 h to stabilize the microstructure at these respective temperatures. The cylinders were then sent for QPA at the Los Alamos Neutron Science Center (LANSCE) in Los Alamos, NM, USA.

### 2.2. Neutron Diffraction

**Figure 1:**
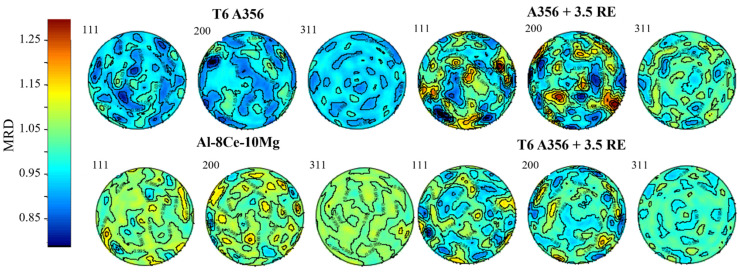
Pole figures of the texture in the FCC-Al matrix of each alloy were assessed in this study before thermal conditioning.

The neutron diffraction experiment was conducted at a short-pulsed spallation neutron source at the LANSCE [44], utilizing the High-Pressure-Preferred Orientation (HIPPO) neutron time-of-flight diffractometer [45,46]. HIPPO utilizes 1200 ^3^He detector tubes arranged on 45 panels, covering 51.7% of 4π by conducting three scans per sample (rotating the sample in between scans about the vertical axis by 0°, 67.5°, and 90°) [46,47]; the latter was employed for this study. Samples were glued on Cd-wrapped sample holders (shielding diffraction from the holder material) and measured for 15 min at 100 μA proton beam current (thus adjusting for possible proton beam fluctuations) per rotation angle. The raw data were processed using the Material Analysis Using Diffraction (MAUD) software (version 2.9993) and the E-WIMV algorithm, which utilized a resolution of 7.5° to derive the orientation distribution function from the diffraction data and calculate pole figures [45,48]. Both texture and phase fraction refinements were conducted to collect an accurate QPA of each material and thermal condition. Figure 1 below shows that the alloys exhibited extremely weak textures (E-WIMV, resolution 7.5°); therefore, random texture was assumed for the phase analysis. The absence of texture shows that reflections do not vary significantly; therefore, the phase analysis can be conducted with high levels of accuracy.

### 2.3. Thermodynamic Modelling in ThermoCalc^TM^

This study utilized ThermoCalc^TM^ equipped with the TCAL9 Al database to determine the phase evolution of all three alloys (A356, A356 + 3.5RE, and Al-8Ce-10Mg) during solidification. Equilibrium and Scheil (nonequilibrium) simulations were performed to understand the effect of solidification rates on the microstructure of each alloy. The equilibrium solidification simulations assume thermal equilibrium at any temperature without considering the effect of time. They provided valuable insight into each alloy composition’s anticipated heat treatment (T6) or thermal conditioning outcomes. The Scheil simulations assume the complete mixing of the liquid, the equilibrium is at the phase boundary between the solid and liquid phases, and there is no back diffusion from the solid to the liquid phases. Scheil solidification simulation explains how nonequilibrium cooling can trap solute in the matrix, creating supersaturation. This information is valuable to this study because when heat treatments or thermal conditioning is applied, these concentrated solute atoms (either in the matrix or in the intermetallics) migrate and alter phase amounts, which is the focus of characterization in this study.

## 3. Results

### 3.1. Thermodynamic Simulations

**Figure 2:**
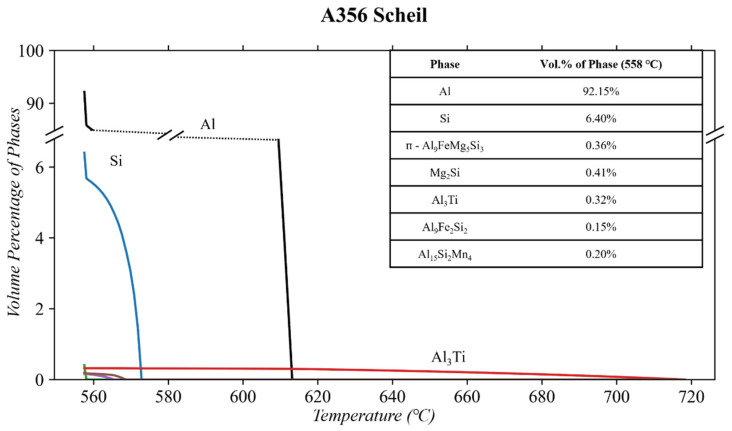
Scheil solidification of the A356 alloy composition using ThermoCalc^TM^.

The Scheil (nonequilibrium) solidification diagrams for the A356 composition from Table 1 are shown in Figure 2. The Scheil diagram shows that the phases of significant volume percentages are Al and Si. In minor quantities, these are the Mg_2_Si, ᴨ-Al_9_FeMg_5_Si_3_, Al_3_Ti, Al_9_Fe_2_Si_2_, and Al_15_Si_2_Mn_4_ phases. At the solidus temperature (shown in the Scheil diagram as 558 °C), excessive amounts of Mg and Si are trapped in a solid solution of the matrix (0.8 and 1.3 wt. %, respectively) due to nonequilibrium cooling.

**Figure 3:**
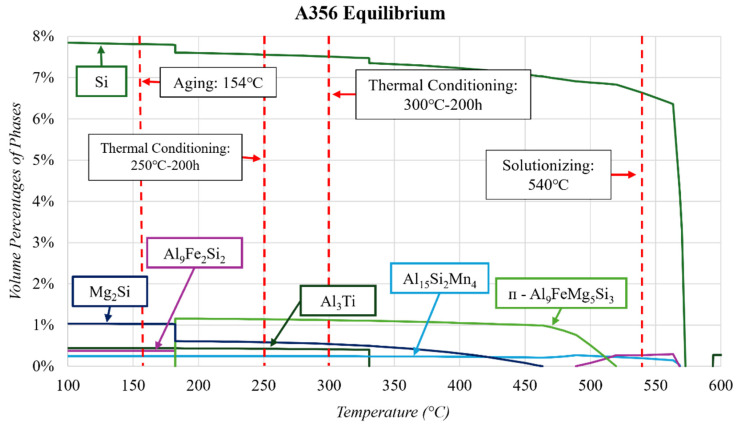
Equilibrium solidification of the A356 alloy composition highlights the thermal conditioning specific to this study.

The equilibrium diagram for the A356 composition is shown in Figure 3. Figure 3 shows that ThermoCalc^TM^ predicts the Si phase will grow from ~6.4 to 7.4 vol.% after the solidus temperature. The equilibrium diagram also predicts that Si, Fe, Mg, and Al begin to precipitate out, starting at ~520 °C, to form the ᴨ-Al_9_FeMg_5_Si_3_ phase. This phase then dissolves at 182 °C and is replaced by the precipitation of the Mg_2_Si phase. These phases were also reported by Stroh et al. [17,18] and Sims [49] and were in good agreement with the equilibrium simulation as opposed to the Scheil after heat treatment. The Scheil and equilibrium ThermoCalc^TM^ simulations also predict the precipitation of Al_9_Fe_2_Si_2_, Al_3_Ti, and Al_15_Si_2_Mn_4_ at low volume percentages (<0.5%). These phases are rarely reported as significant in the published literature on A356.

The thermal treatments of interest in this study are the T6 and conditioning at 250 and 300 °C. Therefore, it is important to highlight what the equilibrium solidification diagrams predict will happen to the microstructure at these temperatures of interest. Figure 3 shows the equilibrium diagram with these temperatures highlighted. As expected, the T6 heat treatment (540 °C solutionizing, quenching, followed by aging at 154 °C) represented in the ThermoCalc^TM^ equilibrium diagram shows that the solutionizing should result in the complete dissolution of the Mg_2_Si, Al_3_Ti, and ᴨ-Al_9_FeMg_5_Si_3_ phases and the partial dissolution of the Si phase. After this, the aging should precipitate out the Mg_2_Si phase (which is well known [11,17,18,49]) and the Al_3_Ti phase (Ti is typically used for grain refinement purposes [14,50], and this phase is not typically reported in the microstructure). The Al_9_Fe_2_Si_2_ phase is also expected to precipitate out of the matrix according to ThermoCalc^TM^, which is much more favourable for mechanical properties than the ᴨ Fe-containing phase [1]. The transition from the Al_9_Fe_2_Si_2_ phase to the ᴨ-Al_9_FeMg_5_Si_3_ phase is expected to happen around 182 °C at the expense of some Mg_2_Si. Stroh et al. [33] confirmed the presence of the ᴨ-Al_9_FeMg_5_Si_3_ phase at approximately 1.2 vol.% after a T6 heat treatment. This volume percentage was estimated by calculating the area of scanning electron microscopy micrographs using ImageJ and correlated well with the ThermoCalc^TM^ prediction of 1.14 vol.% between 182 and 400 °C. An Al matrix and a Si eutectic phase were also discussed in the study by Stroh et al., but specific values of their volume percentages were not given.

The equilibrium diagrams in Figure 2 and Figure 3 show that these phases (Mg_2_Si and Si) will partially dissolve into the matrix or form other phases when thermal conditioning is applied at 250 or 300 ℃. The Mg_2_Si and Si phases are heavily relied upon for their strengthening benefits to the A356 alloy [14,49,51]. However, excess Si is likely trapped in the matrix as a solid solution due to the nonequilibrium cooling. When conditioning is applied, Si will migrate out of the solution, resulting in the increases in the Mg_2_Si and Si phases [11,49].

**Figure 4:**
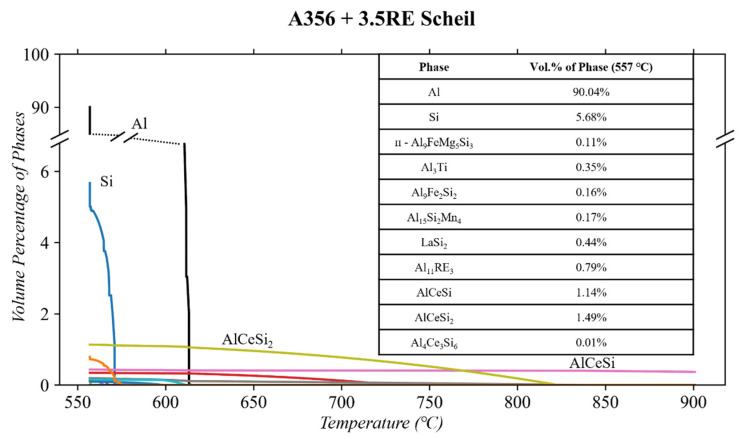
Scheil solidification of the A356 + 3.5RE alloy composition using ThermoCalc^TM^.

Adding 3.5% RE to the A356 alloy constitutes a more complex microstructure. Figure 4 shows both the equilibrium and Scheil solidification diagrams. The addition of RE mischmetal results in five additional phases compared to A356: AlCeSi, AlCeSi_2_, Al_4_Ce_3_Si_6_, LaSi_2_, and Al_11_RE_3_. The solidus temperature (shown in the Scheil diagrams) is 557 °C, similar to the T6 A356 shown in Figure 2. Similar amounts of Mg and Si (0.8 and 1.3 wt. %, respectively) are trapped in a solid matrix solution compared to A356.

Comparatively, the equilibrium diagram is shown in Figure 4. The equilibrium simulations predict the Si phase will grow from ~5.5 to 7.0 vol.% after the solidus temperature to 100 °C. These phases were also reported by Stroh et al. [17,18] and Sims [49] and were in good agreement with the equilibrium simulation as opposed to the Scheil after heat treatment. The Stroh et al. [17] reported no presence of the LaSi_2_ and Al_11_RE_3_. This was attributed to the large presence of La in the AlSiRE [1]. The AlSiRE phase presented in [1] and [14] is characterized as similar to the τ1 phase presented in [5] and [21]. These phases could be any combination of the three AlCeSi phases presented by the ThermoCalc^TM^ simulations. Aghaie et al. [31], in a B319 + Ce alloy, found the presence of both AlCeSi_2_ and Al_4_Ce_3_Si_6_. In the equilibrium solidification diagram, AlCeSi transitions to AlCeSi_2_ at 611 °C. Shortly after this, the AlCeSi_2_ phase transitions to Al_4_Ce_3_Si_6_ at 598 °C. Based on the EDS compositional analysis in [1], it is likely that the Al_4_Ce_3_Si_6_ is the τ1 phase highlighted within these studies. The high solubility of La in this phase, combined with its high thermal stability, suggests that the Al_11_RE_3_ phase ThermoCalc^TM^ predicts will also be absent in the microstructure of the as-cast and T6 alloy. It was reported that in the as-cast state, the A356 + 3.5RE alloy consisted of 4.37% AlSiRE, 2.00% Mg_2_Si, 1.85% ᴨ-Al_9_FeMg_5_Si_3_, and 0.70% Al_20_Ti_2_RE (all in vol.%). The Al_20_Ti_2_RE phase is not predicted in any ThermoCalc^TM^ simulation; however, it is well documented in most Al-RE alloys containing Ti as a grain refiner [52,53,54,55].

**Figure 5:**
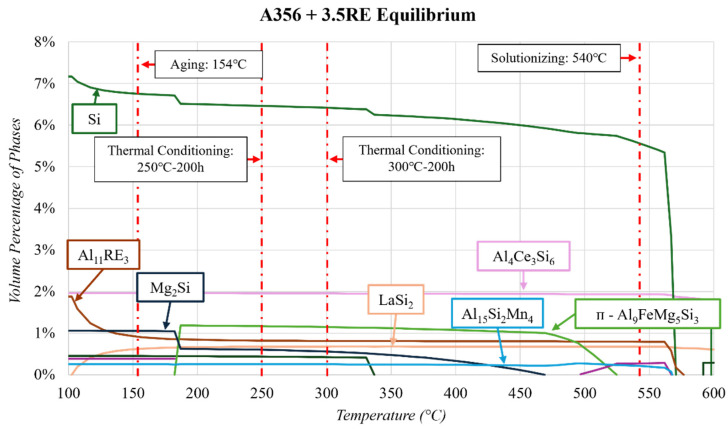
Equilibrium solidification of the A356+ 3.5RE alloy composition highlights the thermal conditioning specific to this study.

Following the principle shown in Figure 3, taking a more in-depth look at the equilibrium diagram for phases at temperatures of interest is paramount. Figure 5 gives a closer look at the aging (154 °C), solutionizing (540 °C), and thermal conditioning (250 and 300 °C) temperatures and the phases predicted. ThermoCalc^TM^ indicates the same transitions for Mg_2_Si, ᴨ-Al_9_FeMg_5_Si_3_, Si, and Al_3_Ti as in regular A356 (Figure 3). It is also similar that the Al_15_Si_2_Mn_4_ phase is present in the equilibrium diagram but not reported in any of the literature for this alloy.

The main difference in the equilibrium solidification of A356 and A356 + 3.5RE is the Al_4_Ce_3_Si_6_ phase (2 vol.%, thermally stable up to 550 °C), the Al_11_RE_3_ phase, and the LaSi_2_ phase. The LaSi_2_ phase is shown to transition to the Al_11_RE_3_ phase between 175 and 100 °C. The vol.% of the Si phase also increases in this temperature range. However, as Stroh et al. [17] report, the formation of these phases is unlikely as there is expected to be a significant amount of La in the solid solution of the Al_4_Ce_3_Si_6_ phase.

**Figure 6:**
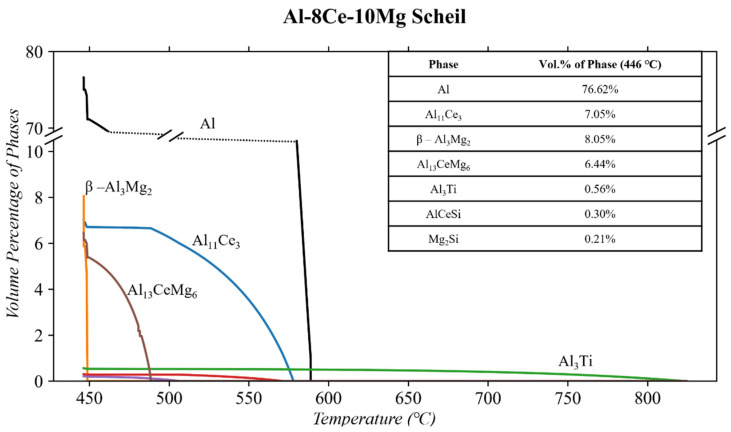
Scheil solidification of the Al-8Ce-10Mg alloy composition using ThermoCalc^TM^.

The last alloy of interest is Al-8Ce-10Mg (detailed composition in Table 1). The Scheil solidification diagrams of this composition are shown in Figure 6. The simulation revealed the presence of the following phases during solidification: Al, Al_11_Ce_3_, β-Al_3_Mg_2_, Al_13_CeMg_6_, Mg_2_Si, AlCeSi, and Al_3_Ti. The latter three phases are in minor quantities (<0.6 vol.%) while the prior four are shown in significant quantities. Of the significant phases, Al begins to precipitate at 587 °C. Shortly thereafter, Al_11_Ce_3_ begins to precipitate at 578 °C. At the solidus temperature (446 °C), ThermoCalc^TM^ predicts ~16.7 wt. % Mg is trapped in the solid solution of the matrix.

**Figure 7:**
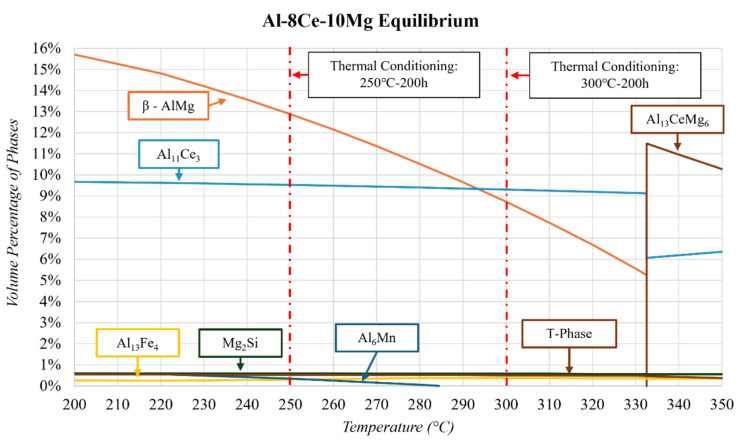
ThermoCalc^TM^ of equilibrium solidification of the Al-8Ce-10Mg alloy composition highlighting the thermal conditioning at 250 and 300 °C.

The alternative equilibrium diagram, showing what potentially would happen after the solidus, is shown in Figure 7. ThermoCalc^TM^ predicts that a ternary Al-Ce-Mg phase (at 332 °C) separates into β-Al_3_Mg_2_ and Al_11_Ce_3_. In all the literature on the Al-8Ce-10Mg alloy, SEM and EDS confirm the presence of Al, Al_11_Ce_3_, and β-Al_3_Mg_2_ [18,40,49,52,56,57]. In no other study were the ternary Al_13_CeMg_6_ and other minor phases found in the microstructure of Al-8Ce-10Mg.

There was no heat treatment used on the Al-8Ce-10Mg alloy in this study. However, 250 and 300 °C thermal conditioning was applied on separate samples for 200 h. Therefore, similar to the other alloys discussed, it is important to closely examine the equilibrium ThermoCalc^TM^ diagram at these respective temperatures. Figure 7 shows the equilibrium diagram between 200 and 350 °C. The diagram shows that as a result of conditioning, the β-AlMg phase is expected to dissolve into the matrix, creating Al with Mg in a solid solution. However, nonequilibrium cooling results in a significant amount of Mg being trapped in a solid solution within the matrix [49]. Therefore, the β-Al_3_Mg_2_ phase is expected to increase due to the Mg in solid solution having a propensity to migrate out and stabilize the β-AlMg phase. This β-Al_3_Mg_2_ phase is proven to be detrimental to mechanical properties [38,49]. These publications do not include specific vol.% of these phases of significance before or after thermal conditioning, making the QPA focus on this study all the more relevant and necessary.

### 3.2. Quantitative Phase Analysis in MAUD

**Figure 8:**
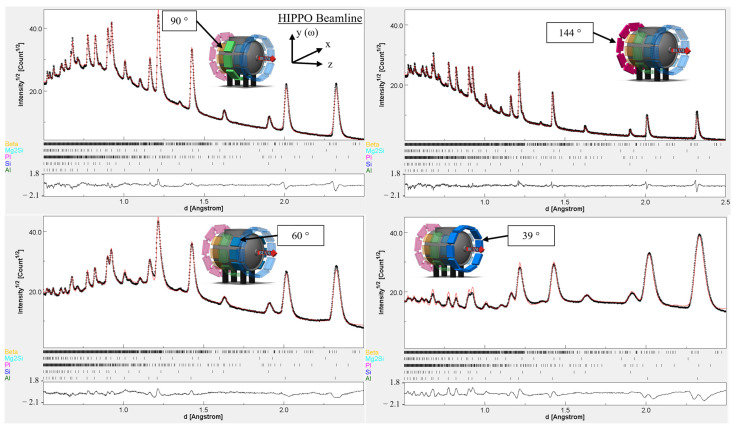
Rietveld refinement fitting of the 39, 60, 90, and 144° panels at 0° rotation of the T6 A356 neutron data using MAUD (2.9993).

To understand the effects of thermal conditioning for specific operating temperatures on the microstructures of T6 A356, A356 + 3.5RE, T6 A356 + 3.5RE, and Al-8Ce-20Mg, a neutron diffraction experiment was carried out using the HIPPO beam at LANSCE and the respective software for Rietveld refinement of diffraction data (MAUD 2.9993). Figure 8 shows the degree of fit targeted in this study. It shows the Rietveld refinement of the 90 (0° rotation) panels of the HIPPO detectors of the T6 A356 alloy. The MAUD setup for each alloy was guided by [43,45,58,59] to ensure a thorough and accurate QPA was conducted since a limited amount of available MAUD studies do not work with multi-phase materials. Phases and their crystalline structures used were CIF files gathered from the crystallography open database [60] and the inorganic crystal structure database, except the Al_11_Ce_3_ phase. Since an appropriate Al_11_Ce_3_ CIF file was not available, one was created using the parameters from [61].

Specifics of the fitting parameters for each MAUD simulation are shown in Table 2. Each simulation had a d-spacing between 0.5 and 3.0 Å. There was an aim to fit sigma values (R_wp_/R_exp_) close to 1 and the R_wp_ values being well below 10%, which are the typical indicators for well-fit Rietveld refinements [43,58,62,63,64].

Table 3 through 5 show the QPA for each alloy and their respective thermal conditioning: Table 3 shows the results of the T6 A356 alloy, Table 4 shows the results of the as-cast and T6 A356 + 3.5RE alloy, and Table 5 shows the results of the Al-8Ce-10Mg alloy.

For the T6 A356 alloy, all the phases found in the ThermoCalc^TM^ simulations (Figure 1 and Figure 2) were included in the MAUD simulation for the Rietveld refinement. Al_3_Ti, Al_9_Fe_2_Si_2_, and Al_15_Si_2_Mn_4_ were quickly excluded during the refinement. Stroh et al. [17] identified the β-Al_5_FeSi phase using SEM and EDS. Therefore, this phase was also included in the Rietveld refinement. Table 3 shows that the β-Al_5_FeSi phase existed in the microstructure of the T6 A356 alloy at low volume percentages (0.5 vol.%). The Al, Si, ᴨ-Al_9_FeSi_3_Mg_5_, and Mg_2_Si phases were all found in volume percentages of 89.1, 7.0, 3.2, and 0.2, respectively.

After thermal conditioning at 250 °C, the alloy experienced an increase in the Si phase (7 to 8 vol.%). This increase comes at the expense of a decrease in every other phase (Al, ᴨ-Al_9_FeSi_3_Mg_5_, Mg_2_Si, and β-Al_5_FeSi). The phases in the T6 A356 alloy responded similarly to both 250 and 300 °C conditioning. Most of the microstructure stabilizes except for Mg_2_Si. Thermally conditioned at 250 °C, the Mg_2_Si phase reduced in volume percentage down 23.37% (from 0.23 to 0.17). However, conditioning at 300 °C slightly reduces Mg_2_Si (0.23 to 0.21). This will be further analyzed later in Section 4 of this paper.

Similar to the discussion on the T6 A356 alloy, the ThermoCalc^TM^ simulation of the composition of the A356 + 3.5RE phases was used for the MAUD Rietveld refinement. The following phases were not found in any significant quantities: AlCeSi, AlCeSi_2_, Al_3_Ti, Mg_2_Si, Al_11_RE_3_, Al_9_Fe_2_Si_2_, Al_15_Si_2_Mn_4_, and LaSi_2_. Also similar to the T6 A356 was the β-Al_5_FeSi phase, identified by Stroh et al. in the A356 + 3.5RE alloy [1,17]. In the same studies, there was also confirmation of an Al-RE-Ti phase identified as Al_20_Ti_2_RE. This phase is reported in another study [52,53] as Al_20_Ti_2_Ce. A CIF file for the Al_20_Ti_2_Ce phase was used in the Rietveld analysis of this alloy. Table 4 shows the results of the MAUD QPA of the as-cast and T6 A356 + 3.5RE alloy. The phase vol.% changes (indicated by the percentages within the brackets) are comparisons between the conditioned and the original unconditioned sample. For example, A356 + 3.5RE 250 °C-200 h values are compared to the unconditioned A356 + 3.5RE phase vol.% values, and the difference is reported in brackets under the conditioned phase value. Similarly, the percent change in the T6 A356 + 3.5RE 250 °C-200 h alloy would be the unconditioned T6 A356 reference sample. This point avoids confusion on which percent change each thermally conditioned alloy references.

Table 4 shows that the Si phase of the A356 + 3.5RE alloy in the as-cast and T6 states both exhibit increases in vol.% with applied thermal conditioning. The Al_4_Ce_3_Si_6_ phase is relatively stable at these temperatures, except for the presence of this phase in the as-cast A356 + 3.5RE 250 °C-200 h alloy, being lower than average (2.07 vol.%). This discrepancy is likely a result of a variation in composition (discussed in more detail in Section 4). The Al_20_Ti_2_Ce and β-Al_5_FeSi phases were also relatively stable when subjected to thermal conditioning. However, after a T6 heat treatment, the Al_20_Ti_2_Ce phase decreased in volume while the β-Al_5_FeSi phase increased. The ᴨ-Al_9_FeSi_3_Mg_5_ phase had a small decrease in volume with increasing thermal conditioning temperatures. This phase also decreased in volume in response to the T6 heat treatment.

Table 5 shows the QPA results from the Al-8Ce-10Mg alloy before and after thermal conditioning. It shows the main trend of an increase in β-Al_3_Mg_2_ ‘pooling’ with thermal conditioning at 250 and 300 °C, similar to chapter 5 in Sims [49]. This increase was likely a coarsening of this phase at the expense of Mg coming out of the solid solution of the matrix, as shown by a slight decrease in the Al phase. The Al_11_Ce_3_ phase also shows a slight decrease in vol.%. However, this phase is quite stable at these thermal conditioning temperatures. Therefore, the difference can be attributed to the slight compositional differences in the material.

## 4. Discussion

### 4.1. T6 A356

**Figure 9:**
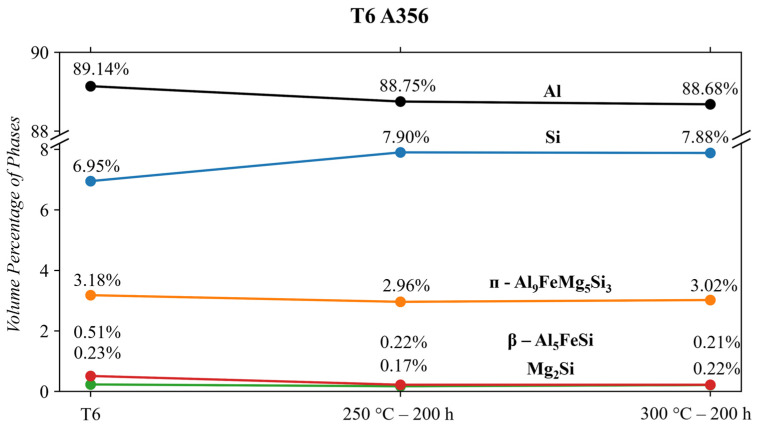
T6 A356 QPA results.

The results from Table 3 are shown in a graphical format in Figure 9 to visualize the vol.% changes in each phase. The ᴨ-Al_9_FeSi_3_Mg_5_ phase was found in much higher volume percentages than predicted in ThermoCalc^TM^ (~3.0 vs. ~1.0). Stroh et al. [14] were among the few attempts to try and quantify the vol.% in a T6 A356 alloy (same composition and heat treatment used in this study). Using SEM micrographs and ImageJ threshold processing, study [14] quantified the ᴨ-Al_9_FeSi_3_Mg_5_ phase to be ~1.2 vol.%, in good correlation with the ThermoCalc^TM^ result. However, as mentioned previously, this processing method can be skewed by aspect ratios of phases and relies heavily on large areas to achieve statistical reliability. Also, determining the proper contrast to distinguish the difference between the ᴨ-Al_9_FeSi_3_Mg_5_ phase and the Si phase using ImageJ can be difficult and subjective. This may lead researchers to favour the ThermoCalc^TM^ values for good agreement and verification. Time-of-flight neutron diffraction, however, is a much more reliable technique for the QPA of bulk phase compositions [43,62]. Therefore, the elevated vol.% of the ᴨ-Al_9_FeSi_3_Mg_5_ phase is likely a more accurate representation of the presence of this phase in the microstructure than the previously used surface techniques.

The QPA results in Figure 9 also show a distinct relationship between the ᴨ-Al_9_FeSi_3_Mg_5_ and Mg_2_Si phase. When the ᴨ-Al_9_FeSi_3_Mg_5_ phase of the T6 A356 alloy decreases in vol.% after 250 °C conditioning, the Mg_2_Si phase does the same. However, this is not a result of thermal conditioning but rather a slight difference in composition between alloys. Wang et al. [65,66] and Taylor et al. [67] identified that the ᴨ-Al_9_FeSi_3_Mg_5_ phase is quite thermally stable when the Mg content in an alloy is above 0.5 wt. % (the Mg content in Table 1 for A356 is 0.49 wt. %). However, after thermal conditioning at 250 °C for 200 h, the ᴨ-Al_9_FeSi_3_Mg_5_ phase in the T6 A356 alloy decreases from 3.17 to 2.96 vol.%. This is also accompanied by a decrease in Mg_2_Si from 0.23 to 0.17. This decrease in Mg_2_Si can be explained by slight compositional differences between alloys. Taylor et al. [67] show that a slight decrease in Mg content below 0.5 wt. % of the alloys would decrease the vol.% Mg_2_Si and ᴨ-Al_9_FeSi_3_Mg_5_, similar to what is shown in Figure 9.

The two phases affected by thermal conditioning are the Si and β-Al_5_FeSi. The β-Al_5_FeSi phase decreases from 0.51 vol.% to 0.22 vol.% when conditioning at either temperature. This partial dissolution is consistent with the findings of Wang et al. [65,66]. Opposite the β-Al_5_FeSi phase partial dissolution is the growing response of the Si phase to thermal conditioning. The refinement of the Si phase after a T6 heat treatment coupled with the solid solution of Si in the Al matrix is well known as a significant contributor to the strength of the A356 alloy. However, during thermal conditioning at 250 and 300 °C, a significant amount of the Si in solid solution is removed, resulting in an extensive increase in this phase (~13% increase). This growth is a significant contributor to the reduction in tensile strength that Sims [49] and Stroh et al. reported [17].

### 4.2. As-Cast and T6 A356 + 3.5RE

**Figure 10:**
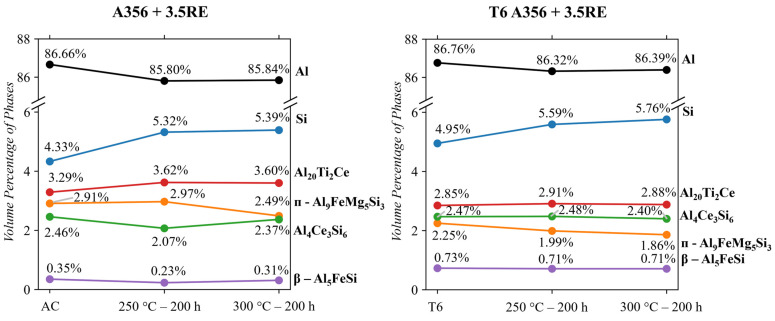
As-cast and T6 A356 + 3.5RE QPA results.

The QPA results for the (as-cast and T6) A356 + 3.5RE alloy are shown in Figure 10. These results are in agreement with Stroh et al.’s paper on this alloy [17] concerning the Mg_2_Si phase. The Rietveld fitting of the diffraction pattern revealed no detectable Mg_2_Si within the alloy. This suggests that the tensile strength of this alloy is independent of Mg_2_Si, unlike its compositionally simpler counterpart, A356. The phases revealed via Rietveld analysis were Al, Si, Al_4_Ce_3_Si_6_ Al_20_Ti_2_Ce, ᴨ-Al_9_FeSi_3_Mg_5_, and β-Al_5_FeSi. After conditioning the alloy at 250 °C for 200 h, the Al_4_Ce_3_Si_6_ phases decreased in vol.% by 34.7. This decrease in vol.% increases the Si phase and slightly increases the Al_20_Ti_2_Ce phase. The Si from the Al_4_Ce_3_Si_6_ and excess trapped in the matrix contribute to the growth of the Si phase. The growth of the Al_20_Ti_2_Ce phase is a result of sharing common elements (Ce and Nd [17]) with the Al_4_Ce_3_Si_6_ phase. During solidification, the Al_20_Ti_2_Ce phase is known to precipitate early as a primary phase [68], similar to Al_4_Ce_3_Si_6_. As a result, they compete for similar elements and nonequilibrium cooling results in both these phases forming in a metastable condition. Thermal conditioning at 250 °C causes the excess Ti from the matrix and Ce and Nd from Al_4_Ce_3_Si_6_ to stabilize the Al_20_Ti_2_Ce phase, resulting in the growth shown A356 + 3.5RE in Figure 10. The ᴨ and β Fe-containing phases remain thermally stable at 250 °C.

At 300 °C conditioning, the ᴨ-Al_9_FeSi_3_Mg_5_ phase partially dissolves, and the Al_4_Ce_3_Si_6_ and β-Al_5_FeSi return to similar vol.% as the as-cast values. The Al_20_Ti_2_Ce and Si phases remain at a similar vol.% as the 250 °C conditioned samples. Increases in the vol.% of the Al_4_Ce_3_Si_6_ and β-Al_5_FeSi phases and decreases in the ᴨ-Al_9_FeSi_3_Mg_5_ phase are typically beneficial for enhancing the mechanical properties of alloys [1,31,69]. However, the increase in the Si and Al_20_Ti_2_Ce phases is shown to be detrimental to mechanical properties [17,53]. At 250 °C in the as-cast state, the A356 + 3.5RE alloy performs similarly to the T6 A356 alloy, showing no mechanical benefit [18] with this microstructural evolution. However, at 300 °C, the as-cast A356 + 3.5RE alloy significantly outperforms the T6 A356 alloy [18], showing that the enhancing benefits of the increase in the Al_4_Ce_3_Si_6_ phase and the decrease in ᴨ-Al_9_FeSi_3_Mg_5_ outweigh the detrimental effect of Si and Al_20_Ti_2_Ce phase increases.

After the T6 heat treatment of the A356 + 3.5RE alloy, the vol.% of the β-Al_5_FeSi increased and the ᴨ-Al_9_FeSi_3_Mg_5_ phase decreased. This transition stabilized the β-Al_5_FeSi phase at ~0.7 vol.%, which was then unaffected by thermal conditioning. The ᴨ-Al_9_FeSi_3_Mg_5_ phase was thermally unstable after the T6 heat treatment and linearly decreased with increasing thermal conditioning (2.25 vol.% to 1.99 vol.% at 250 °C then 1.86 vol.% at 300 °C). This lack of thermal stability of the ᴨ-Al_9_FeSi_3_Mg_5_ can be linked to Tayor et al.’s [67] findings regarding the limited mobility of Mg. Stroh et al. [1,17] found Mg was present in the Al_20_Ti_2_RE, AlSiRE, and β-Al_5_FeSi phases, which are thermally stable and unaffected by thermal conditioning. Therefore, the Mg within these phases cannot migrate and stabilize the ᴨ-Al_9_FeSi_3_Mg_5_ phase [67]; hence, its vol.% decreases when thermal conditioning is applied to the alloy.

The Al_20_Ti_2_Ce phase also decreases in volume after the T6 heat treatment by 13% (from 3.3 to 2.9 vol.%). There is no knowledge of the stability of the Al_20_Ti_2_Ce phase above 400 °C. However, in this instance, solution annealing at 538 °C for 8 h results in the partial dissolution of this phase with the benefit of increasing the more favourable Al_4_Ce_3_Si_6_ phase. After a T6 heat treatment, the Al_20_Ti_2_Ce phase is relatively unaffected by thermal conditioning (i.e., thermally stabilized) unlike the as-cast state of the alloy, which showed that thermal conditioning caused slight growth of this phase. This phase’s reduction and stabilization, the decreased vol.% of the ᴨ-Al_9_FeSi_3_Mg_5_ phase, and the increased vol.% of the β-Al_5_FeSi phase are large contributors to explain why the mechanical properties Stroh et al. [17,18] reported at 250 and 300 °C are significantly better than the as-cast state of the same alloy.

The one constant between the as-cast and T6 A356 + 3.5RE alloys is the growth of the Si phase when the alloys are subjected to thermal conditioning. The Si phase of the T6 A356 alloy also experienced a similar vol.% increase when subjected to thermal conditioning. However, the A356 + 3.5RE and T6 A356 + 3.5RE both had better tensile properties than the T6 A356 alloy [17], despite this similar Si growth. The refinement of this Si phase from an irregular blocky intermetallic to spheroidized particles after a T6 heat treatment is well known to enhance the room temperature strength of A356-based alloys [14,18]. However, the growth of this phase above 200 °C limits its potential for automotive powertrain applications. The A356 + 3.5RE suggests that both scenarios combine the best approaches, utilizing the refined Si phase for room-temperature strength and the RE intermetallics for elevated-temperature benefits. These RE intermetallics, in addition to the Fe- and Si-containing phases, respond well to a conventional T6 heat treatment, which ultimately leads to enhanced tensile strength [1,17], even after the microstructure stabilizes thermal conditioning. The results from this QPA study provide a complete analysis of how the fundamental microstructural evolution results in these elevated tensile properties.

### 4.3. As-Cast Al-8Ce-10Mg

**Figure 11:**
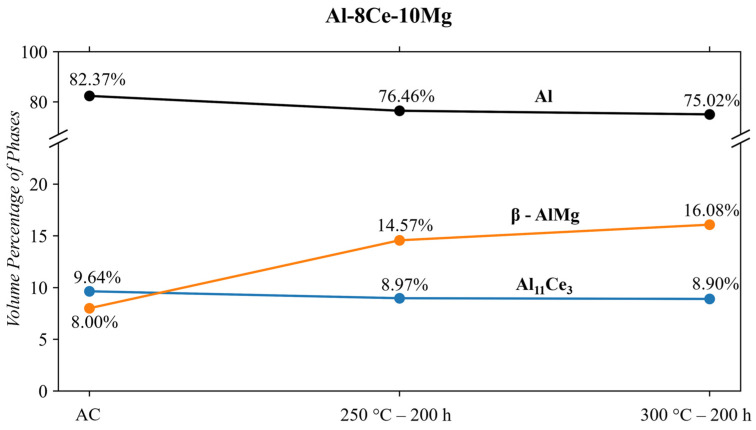
Al-8Ce-10Mg QPA results.

The results of the Al-8Ce-10Mg alloy MAUD QPA are shown in Figure 11. In the as-cast state, there is an excessive amount of Mg in the matrix as a solid solution, identifiable by the high vol.% of Al and low amounts of β-Al_3_Mg_2_. The Scheil solidification in ThermoCalc^TM^ (Figure 6) predicted the excess Mg in the solid solution to be ~16 wt. %. When 250 °C thermal conditioning is applied, the β-Al_3_Mg_2_ phase grows by 82% from 8.0 to 14.6 vol.%. The growth of the β-Al_3_Mg_2_ phase is due to the high amount of Mg in the alloy. Golovin et al. [70] show that alloys with an excess of 8 wt. % Mg have an absence of internal friction that inhibits the migration of Mg, which is the case in this alloy. This explains why thermal conditioning results in the extensive growth of the β-Al_3_Mg_2_ phase, shown in Figure 11. Sims [49] correlates the increase in the β-Al_3_Mg_2_ phase to a decrease in mechanical properties. In the same study, Sims observes the β-Al_3_Mg_2_ to dissolve back into the matrix around 400 °C after extensive thermal conditioning (>500 h). The 300 °C-200 h thermal conditioning targeted in this study shows that the β-AlMg phase continues to grow at 300 °C up to 16.1 vol.% (doubled from the as-cast state).

The other significant phase identified in the Rietveld analysis is the Al_11_Ce_3_ phase. This phase appears relatively stable in both this study and the one by Sims [49]. The small volume percentage discrepancy of the Al_11_Ce_3_ phase can be attributed to slight compositional variances. The other phases predicted by ThermoCalc^TM^ (Al_13_CeMg_6_, Mg_2_Si, Al_13_Fe_4_, Al_6_Mn, and T-phase) were not identified in the MAUD Rietveld analysis. The microstructural evolution of this alloy suggests that the 250 to 300 °C range is not an ideal operating temperature for this alloy. It is not until 400 °C that the microstructure stabilization becomes beneficial for mechanical properties [49].

## 5. Conclusions

This study aimed to conduct an in-depth quantitative phase analysis via the time-of-flight neutron diffraction of a conventional T6 A356 alloy and advanced A356 + 3.5RE and Al-8Ce-10Mg alloys. These alloys were subjected to 250 and 300 °C thermal conditioning to understand how the phases of all three alloys stabilized at these temperatures.

- The T6 A356 alloy contained the following phases, confirmed via Rietveld analysis: cubic Al, Si, Mg_2_Si, ᴨ-Al_9_FeSi_3_Mg_5_, β-Al_5_FeSi. Conditioning at 250 and 300 °C decreases the β-Al_5_FeSi phase from 0.51 to 0.22 vol.%. The Si phase grows significantly by ~13% when subjected to either condition temperature. The volume of other phases remained relatively stable when subjected to thermal conditioning. The growth of the Si phase significantly weakens this alloy and makes it appealing for this temperature region.
- The A356 + 3.5RE alloy responded to 250 °C conditioning with an increase in the Si, ᴨ-Al_9_FeSi_3_Mg_5_, and Al_20_Ti_2_Ce phases, causing a reduction in Al_4_Ce_3_Si_6_. When subjected to 300 °C thermal conditioning, the Al_4_Ce_3_Si_6_ and β-Al_5_FeSi stabilize, the Si and Al_20_Ti_2_Ce phases increase similarly to the 250 °C condition, and the ᴨ-Al_9_FeSi_3_Mg_5_ partially dissolves. The benefits of increasing the Al_4_Ce_3_Si_6_ and decreasing the ᴨ-Al_9_FeSi_3_Mg_5_ are beneficial for mechanical properties. However, the growth effects of Si and Al_20_Ti_2_Ce are typically detrimental to mechanical properties.
- When subjecting the A356 + 3.5RE alloy to the same T6 heat treatment as the A356 alloy, the Al_4_Ce_3_Si_6_ phase stabilizes, the Al_20_Ti_2_Ce decreases by ~13% from 3.3 to 2.9 vol.%, and the volume of β-Al_5_FeSi doubles from 0.35 to 0.7 vol.%. These phases become thermally stable and have negligible responses to thermal conditioning at 250 and 300 °C. The ᴨ-Al_9_FeSi_3_Mg_5_ phase decreases by 23% from 2.91 to 2.25 vol.%. This phase is thermally unstable due to the lack of Mg mobility and decreases by ~12% and 18% in response to thermal conditions at 250 and 300 °C, respectively. The volume of the Si phase within the alloy increases similarly to the T6 A356 and the as-cast A356 + 3.5RE alloys. All these refinements in the phases explain why the T6 A356 + 3.5RE alloy significantly outperforms the T6 A356 alloy in the desirable temperature region in other studies.
- The phases of the Al-8Ce-10Mg alloy in the as-cast state consist of Al (with Mg in solid solution), β-Al_3_Mg_2_, and Al_11_Ce_3_. The Al_11_Ce_3_ phase changes negligibly in response to thermal conditioning at 250 and 300 °C. The β-AlMg phase grows by 82% at 250 °C, and its volume doubles at 300 °C. This growth results from the alloy having little internal resistance to the migration of Mg from the solid solution with the matrix, evidenced by the decrease in the Al phase in response to the thermal conditions. The benefits from the stable Al_11_Ce_3_ are negatively affected by the increase in β-Al_3_Mg_2_ at 250 and 300 °C, making utilizing the alloy at this temperature region unfavourable. The full benefits of the stable Al_11_Ce_3_ phase will only be obtained at the temperature point where it is more favourable for the β-Al_3_Mg_2_ to dissolve back into the matrix.

As Al alloys become a more prominent solution to problems associated with elevated-temperature applications, it is paramount that they be characterized in a manner that suits the application conditions. Not thermally conditioning alloys before testing them at elevated temperatures results in misrepresenting the alloy’s microstructure at these temperatures. An unrepresentative microstructure leads to inaccurate tensile data, which can lead to much more significant engineering issues. This study indicates that thermal conditioning is important to establish quality microstructures, especially when developing novel Al alloys for high-temperature applications.

## Figures and Tables

**Table 1:** Alloy compositions of A356, A356 + 3.5RE, and Al-8Ce-10Mg in weight percentage (wt. %).

Alloys	Al	Si	Mg	Ce	Cu	Fe	Mn	Ti	La	Nd	Pr
A356	Bal.	7.28	0.49	-	0.03	0.13	0.10	0.20	-	-	-
A356 + 3.5RE	Bal.	7.28	0.49	1.83	0.03	0.13	0.10	0.20	0.92	0.58	0.19
Al-8Ce-10Mg	Bal.	0.15	9.5–10.00	7.84–8.16	0.03	0.15	0.25	0.25	-	-	-

**Table 2:** MAUD parameters and fitting evaluation of the T6 A356 alloy.

Alloy	Conditioning	Sigma	R_wp_ (%)	R_exp_ (%)
A356	T6	1.09	5.92	5.43
A356	T6 + 250 °C-200 h	1.01	5.54	5.49
A356	T6 + 300 °C-200 h	1.06	5.74	5.42
A356 + 3.5RE	As-cast	1.11	6.00	5.41
A356 + 3.5RE	250 °C-200 h	1.06	5.83	5.50
A356 + 3.5RE	300 °C-200 h	1.03	5.51	5.37
A356 + 3.5RE	T6	1.02	5.45	5.36
A356 + 3.5RE	T6 + 250 °C-200 h	1.00	5.52	5.54
A356 + 3.5RE	T6 + 300 °C-200 h	1.04	5.70	5.48
Al-8Ce-10Mg	As-cast	0.95	5.33	5.61
Al-8Ce-10Mg	250 °C-200 h	0.94	5.16	5.49
Al-8Ce-10Mg	300 °C-200 h	0.99	5.51	5.57

**Table 3:** Quantitative phase analysis (QPA) results for the T6 A356 alloy.

Alloy	Conditioning	Volume Percentage of Phases				
		Al	Si	ᴨ-Al_9_FeSi_3_Mg_5_	Mg_2_Si	β-Al_5_FeSi
A356	T6	89.14	6.95 ± 0.02	3.17 ± 0.05	0.23 ± 0.02	0.51 ± 0.02
A356	T6 + 250 °C-200 h	88.75	7.90 ± 0.02	2.96 ± 0.04	0.17 ± 0.03	0.22 ± 0.03
A356	T6 + 300 °C-200 h	88.68	7.88 ± 0.03	3.02 ± 0.05	0.21 ± 0.02	0.22 ± 0.02

**Table 4:** QPA results for the as-cast and T6 A356 + 3.5RE alloy.

Alloy	Conditioning	Volume Percentage of Phases					
		Al	Si	Al_4_Ce_3_Si_6_	ᴨ-Al_9_FeSi_3_Mg_5_	Al_20_Ti_2_Ce	β-Al_5_FeSi
A356 + 3.5RE	As-Cast	86.66	4.33 ± 0.03	2.46 ± 0.09	2.91 ± 0.05	3.29 ± 0.07	0.35 ± 0.05
A356 + 3.5RE	250 °C-200 h	85.80	5.32 ± 0.02	2.07 ± 0.08	2.97 ± 0.05	3.62 ± 0.06	0.23 ± 0.04
A356 + 3.5RE	300 °C-200 h	85.84	5.39 ± 0.02	2.37 ± 0.08	2.49 ±0.05	3.60 ± 0.07	0.31 ± 0.04
A356 + 3.5RE	T6	86.76	4.95 ± 0.02	2.47 ± 0.02	2.25 ± 0.04	2.85 ± 0.02	0.73 ± 0.01
A356 + 3.5RE	T6 + 250 °C-200 h	86.32	5.59 ± 0.01	2.48 ± 0.02	1.99 ± 0.04	2.91 ±0.02	0.71 ± 0.01
A356 + 3.5RE	T6 + 300 °C-200 h	86.39	5.76 ± 0.03	2.40 ± 0.04	1.86 ± 0.03	2.88 ± 0.01	0.71 ± 0.02

**Table 5:** QPA results for the as-cast and thermally conditioned Al-8Ce-10Mg alloy.

Alloy	Conditioning	Volume Percentage of Phase		
		Al	Al_11_Ce_3_	β-Al_3_Mg_2_
Al-8Ce-10Mg	As-Cast	82.4	9.6 ± 0.07	8.0 ± 0.03
Al-8Ce-10Mg	250 °C-200 h	76.5	9.0 ± 0.05	14.6 ± 0.04
Al-8Ce-10Mg	300 °C-200 h	75.0	8.9 ± 0.06	16.1 ± 0.03

## Data Availability

The raw and processed data required to produce these findings are available for download from Mendeley Data: Kozakevich, Jordan (2024), “A Quantitative Phase Analysis by Neutron Diffraction of Conventional and New-Age Aluminum Alloys Thermally Conditioned for Elevated Temperature Applications”, Mendeley Data, V1, https://data.mendeley.com/datasets/m7wzh86cd7/1, accessed 2 July 2024.
